# The impact and inflammatory characteristics of SARS-CoV-2 infection during ovarian stimulation on the outcomes of assisted reproductive treatment

**DOI:** 10.3389/fendo.2024.1353068

**Published:** 2024-04-25

**Authors:** Xiaoling Hu, Guofang Feng, Qichao Chen, Yimiao Sang, Qingqing Chen, Sisi Wang, Shuangying Liu, Long Bai, Yimin Zhu

**Affiliations:** ^1^ Department of Reproductive Endocrinology, Women’s Hospital, School of Medicine, Zhejiang University, Hangzhou, Zhejiang, China; ^2^ Key Laboratory of Reproductive Genetics, Ministry of Education, Zhejiang University, Hangzhou, China

**Keywords:** COVID-19, ovarian stimulation, assisted reproductive, embryo transfer, pregnancy, cytokines

## Abstract

**Introduction:**

Despite the global prevalence of coronavirus disease 2019 (COVID-19), limited research has been conducted on the effects of SARS-CoV-2 infection on human reproduction. The aims of this study were to investigate the impact of SARS-CoV-2 infection during controlled ovarian stimulation (COS) on the outcomes of assisted reproductive treatment (ART) and the cytokine status of patients.

**Methods:**

This retrospective cohort study included 202 couples who received ART treatment, 101 couples infected with SARS-CoV-2 during COS and 101 matched uninfected couples. The parameters of ovarian stimulation and pregnancy outcomes were compared between the two groups. The All-Human Inflammation Array Q3 kit was utilized to measure cytokine levels in both blood and follicular fluid.

**Results:**

No difference was found in the number of good-quality embryos (3.3 ± 3.1 vs. 3.0 ± 2.2, *P* = 0.553) between the infected and uninfected groups. Among couples who received fresh embryo transfers, no difference was observed in clinical pregnancy rate (53.3% vs. 51.5%, *P* = 0.907). The rates of fertilization, implantation, miscarriage, ectopic pregnancy and live birth were also comparable between the two groups. After adjustments were made for confounders, regression models indicated that the quality of embryos (B = 0.16, *P* = 0.605) and clinical pregnancy rate (*P* = 0.206) remained unaffected by SARS-CoV-2 infection. The serum levels of MCP-1, TIMP-1, I-309, TNF-RI and TNF-RII were increased, while that of eotaxin-2 was decreased in COVID-19 patients. No significant difference was found in the levels of cytokines in follicular fluid between the two groups.

**Conclusion:**

Asymptomatic or mild COVID-19 during COS had no adverse effects on ART outcomes. Although mild inflammation was present in the serum, it was not detected in the follicular fluid of these patients. The subsequent immune response needs further investigation.

## Introduction

Coronavirus disease 2019 (COVID-19), is a respiratory illness that spreads easily. It is caused by the severe acute respiratory syndrome coronavirus 2 (SARS-CoV-2), which is highly contagious (1). COVID-19 spread rapidly worldwide after the virus is identified (2). Approximately 80% of infected patients do not show any symptoms or are asymptomatic, while 15% to 20% have severe pneumonia or multiple organ failure (3). Due to the unpredictable impact of SARS-CoV-2 on reproduction, it was recommended to delay assisted reproductive treatment (ART) during the initial phase of the pandemic (4). As a result of China’s relaxation of COVID-19 pandemic control measures since December 2022, more infected patients are during ART treatment being observed (5).

Theoretically, any tissue that expresses the cellular receptor angiotensin-converting enzyme 2 (ACE2) has the potential to be the target for SARS-CoV-2, since it enters host cells primarily via ACE2 (6, 7). Damage is likely to occur in sertoli and leydig cells in the testis, ovarian tissue, oocytes, embryos and endometrium due to the presence of the ACE2 receptor (4, 8–11). The immune system’s response to SARS-CoV-2 infection relies on the alterations of cytokines (12). Patients with severe COVID-19 have elevated levels of proinflammatory cytokines (12–14). Cytokines play important roles in folliculogenesis, oocyte maturation, ovulation, fertilization, embryo development and pregnancy establishment (15, 16). They are also involved in the regulation of steroidogenesis (17). These findings suggest that aberrant inflammary status might impair fertility. Therefore, it is crucial to understand whether the virus affects reproduction. However, despite the global prevalence of this disease, limited research has been conducted on the impacts of SARS-CoV-2 infection on human reproduction. More research is required to address this concern.

The aims of this study were to evaluate the effect of SARS-CoV-2 infection on the embryos quality and pregnancy rates in patients undergoing ART treatment and to explore whether specific cytokines can affect the ART treatment outcomes of patients with SARS-CoV-2 infection to also explored.

## Materials and methods

### Study population

This retrospective study included patients who visited our assisted reproductive unit due to infertility for various reasons and underwent ART treatment from November 1, 2022 to January 31, 2023. Following the onset of the pandemic, all couples undergoing ART treatment were tested for SARS-CoV-2 infection using nasopharyngeal swabs prior to commencing the treatment and the day before oocyte retrieval. Nasopharyngeal swab screening was also conducted on couples who exhibited signs of SARS-CoV-2 infection while undergoing ovarian stimulation or who were exposed to infected individuals and had a high risk of SARS-CoV-2 infection. Nucleic acid or antigen tests were used to diagnose SARS-CoV-2 infection. If one partner who was asymptomatic or mildly symptomatic tested positive for SARS-CoV-2 before oocyte retrieval, the couples decided whether to proceed with the planned oocyte retrieval and/or embryo transfer (ET) or cancel the cycle. COVID-19 patients with moderate or severe infections were advised to cancel their ART treatment cycles. The diagnosing and treating individuals with COVID-19 relied on trial version 9 of the Pneumonia Diagnosis and Management Plan for Novel Coronavirus Infection. Mild COVID-19 was characterized by mild fever, cough, body aches, and other symptoms with no pneumonia; the moderate disease presented with mild pneumonia and other symptoms; and severe COVID-19 was characterized by severe pneumonia and a lack of oxygen. During the study period, none of the couples presented with the severe COVID-19 or required hospitalization. All *in vitro* fertilization (IVF) procedures were conducted in a separate area for individuals who had a positive SARS-CoV-2 RNA test. Additionally, the embryos were stored in a distinct tank filled with liquid nitrogen for cryopreservation. The study group (the SARS-CoV-2-positive group) consisted of couples with at least one SARS-CoV-2-positive partner. The infected group was matched 1:1 by age and cause of infertility to the uninfected group of patients who underwent ART treatment during the same period (SARS-CoV-2-negative group); among these patients neither partner was infected with SARS-CoV-2. A diminished ovarian reserve (DOR) was indicated when the anti-Müllerian hormone (AMH) level was below 1.1 ng/ml (18).

### Treatment protocols

The ovarian stimulation protocols were performed as previously described and categorized into antagonist protocols, long protocols and other protocols, including progestin-primed ovarian stimulation and mild stimulation protocols (19). The stimulation protocols and fertilization methods were chosen based on patient characteristics and past cycle performance. The retrieval of oocytes was performed between 36 and 38 hours following the injection of human chorionic gonadotropin (hCG). The peripheral blood and follicular fluid of females were collected on the day of oocyte retrieval. The follicular fluids were obtained during oocyte retrieval as previously described (20). Most fresh ETs were cancelled due to SARS-CoV-2 infection, and a few asymptomatic patients underwent fresh ET with thorough medical counselling. Fresh ET was performed on Day 3 with the best 1 or 2 embryos. Otherwise, fresh ET was cancelled due to fluid accumulation in the cavity, unfavourable endometrium, the risk of ovarian hyperstimulation, hydrosalpinx, and the need for genetic analysis or surrogacy. On Day 3, embryos that had at least 6 blastomeres with ≤25% fragmentation were categorized as good-quality embryos according to the Istanbul consensus workshop (21). Data on the baseline characteristics and treatment outcome information of the patients were retrieved from the ART data system.

The research was carried out in accordance with the principles of the Helsinki Declaration. The Ethics Committee of the Women’s Hospital, School of Medicine, Zhejiang University (IRB-20230204-R) granted approval for this study, and all subjects provided informed consent.

### Outcome measures

The primary measures were the quantity of good-quality embryos and the rate of clinical pregnancy. The secondary outcome measures were the number of retrieved oocytes, and the rates of fertilization, implantation, miscarriage and live birth. To study the effect of SARS-CoV-2 infection on male sperm quality, semen parameters before and on the day of oocyte retrieval after SARS-CoV-2 infection were compared.

Clinical pregnancy was defined as the presence of one or more gestational sacs, as visualized by transvaginal ultrasound examination. The fertilization rate by IVF was calculated by dividing the number of fertilized oocytes by the number of retrieved oocytes. The rate of fertilization by intracytoplasmic sperm injection (ICSI) was calculated by dividing the number of fertilized oocytes by the number of metaphase II oocytes. The implantation rate was calculated by dividing the number of gestational sacs by the number of transferred embryos. Miscarriage was defined as the loss of an intrauterine pregnancy before gestational week 28. A live delivery was defined as delivery of a newborn at or after 28 weeks of gestation.

### Serum and follicular fluid cytokine measurements

The levels of cytokines in both serum and follicular fluid were measured using the All Human Inflammation Array Q3 kit (RayBiotech Life, GA, USA) following the manufacturer’s instructions. The procedures were performed as previously described (5).

### Statistical analysis

Statistical analysis was performed by using SPSS version 26.0. The concentrations of the quantified inflammatory factors were processed using R version 4.3.2. Quantitative variables with a normal distribution and homogenous variance are expressed as the mean ± standard deviation, and the means were compared using Student’s t test. Categorical variables are summarized using percentages and counts. Differences in the rates were compared by mean of the χ^2^ test. Statistical significance was defined as by a two-sided *P* value less than 0.05.

To determine the factors associated with the quantity of good-quality embryos, a linear regression model was utilized. In the preliminary model, the included variables were age, duration of infertility, cause of infertility, AMH level, the number of retrieved oocytes, and SARS-CoV-2 status. The forward elimination method was utilized to choose the best model, allowing inclusion at P < 0.05 and exclusion at *P* > 0.15. The model was compelled to incorporate of SARS-CoV-2 status. The ultimate model included SARS-CoV-2 status, the number of retrieved oocytes, the AMH level, and the duration of infertility.

Multivariate logistic regression analysis was used to compare the pregnancy rate adjusted for confounding factors, such as female age, duration of infertility, type and causes of infertility, AMH level, number of retrieved oocytes, number of high-quality embryos and number of transferred embryos. The model was compelled to incorporate the SARS-CoV-2 status. We used the forward elimination method to select the optimal model, allowing inclusion when *P* < 0.05 and exclusion when *P* > 0.15. The ultimate model incorporated the SARS-CoV-2 status, infertility type, and the quantity of high-quality embryos.

## Results

### Comparison of baseline characteristics

In total, 101 couples (42 in which the female partner was infected, 23 in which the male partner was infected and 36 in which both partners was infected) met the inclusion criteria and were matched to 101 control couples according to female age and cause of infertility who were not affected by SARS-CoV-2. The mean ages of the females in the study and control groups were comparable, as were the male ages, AMH levels, and BMIs. There were no differences in the type of infertility, infertility cause, number of previous IVF procedures, rate of female vaccination, and rate of male vaccination. The data were shown in **Table 1**. The interval between the time of the last vaccination and the date of the subsequent IVF treatment cycle was 6-24 months.

**Table 1:** Baseline characteristics of ART patients in the SARS-CoV-2-positive versus the control group.

Characteristic	SARS-CoV-2 positive (n = 101)	SARS-CoV-2 negative (n = 101)	*P* value
Female age (years)	33.0 ± 4.5	33.0 ± 4.5	0.937
Male age (years)	34.7 ± 5.9	34.1 ± 5.2	0.383
Duration of infertility (years)	3.4 ± 2.8	3.2 ± 2.5	0.565
AMH (IU/L)	3.2 ± 2.7	2.8 ± 2.1	0.069
BMI (kg/m^2^)	21.4 ± 2.6	21.8 ± 2.6	0.853
Type of infertility			0.067
Primary infertility	46.5% (47/101)	59.4% (60/101)	
Secondary infertility	53.5% (54/101)	40.6% (41/101)	
Causes of infertility			0.998
Tubal	29.7% (30/101)	29.7% (30/101)	
Male	13.9% (14/101)	13.9% (14/101)	
DOR	30.7% (31/101)	29.7% (30/101)	
Others	25.7% (26/101)	26.7% (27/101)	
Previous cycles	1.7 ± 1.2	1.9 ± 1.4	0.557
Female vaccination rate	21.8% (22/101)	19.8% (20/101)	0.729
Male vaccination rate	22.8% (23/101)	20.8% (21/101)	0.733

The data are presented as the means ± standard deviations or percentages and counts. ART, assisted reproductive technology; AMH, anti-Müllerian hormone; BMI, body mass index; DOR, diminished ovarian reserve.

### Comparison of ovarian stimulation cycle characteristics

The cycle characteristics of patients in both the study and control groups, including the treatment protocols, gonadotrophin dosage, the duration of stimulation, the peak E_2_ levels and fertilization methods, were comparable. The quantity of good-quality embryos, the number of oocytes retrieved, the number of fertilized oocytes with 2 pronuclei, the rate of fertilization, and the number of transferred embryos were also comparable between the two groups. The data were presented in **Table 2**. The effects of SARS-CoV-2 infection on semen parameters were further analysed. Among 59 males with SARS-CoV-2 infection, the semen parameters of 50 males whose semen samples were freshly ejaculated by masturbation were compared before and on the day of oocyte retrieval after infection. The semen parameters of the males were summarized in **Supplementary Table S1**. After infection, the progressive motility and complete motility of the sperm did not significantly differ (*P* > 0.05). Although the semen volume, sperm concentration and total sperm count were decreased (*P* < 0.05 for all), the values were still within the normal reference range, and the fertilization method was not influenced by the infection. A linear regression model demonstrated no effect of SARS-CoV-2 infection on the number of good-quality embryos (B = 0.16, *P* = 0.605), whereas the duration of infertility (B = -0.153, *P* = 0.009) and the number of oocytes retrieved (B = 0.135, *P* < 0.001) remained significant factors (**Table 3**).

**Table 2:** Cycle characteristics and treatment outcomes of ART patients in the SARS-CoV-2-positive group versus the control group.

Variable	SARS-CoV-2 positive (n = 101)	SARS-CoV-2 negative (n = 101)	*P* value
COS protocol			0.787
Long protocol	26.7% (27/101)	22.8% (23/101)	
Antagonist protocol	44.6% (45/101)	45.5% (46/101)	
Other protocol	28.7% (29/101)	31.7% (32/101)	
Dosage of Gn used (IU)	2106.9 ± 1036.4	2245.9 ± 851.3	0.050
Duration of stimulation (d)	9.5 ± 3.3	10.2 ± 4.0	0.460
Peak E_2_ level (pmol/L)	9677.7 ± 8030.2	9996.0 ± 7872.9	0.606
Number of oocytes retrieved	9.9 ± 6.8	9.7 ± 6.5	0.391
Fertilization method			0.641
IVF	69.5% (66/95)	66.3% (63/95)	
ICSI	30.5% (29/95)	33.7% (32/95)	
IVF fertilization rate	60.8% (399/656)	62.8% (402/640)	0.461
ICSI fertilization rate	68.4% (128/187)	63.8% (104/163)	0.359
Number of 2PN fertilized oocytes	5.5 ± 5.2	5.4 ± 5.2	0.02
Number of good-quality embryos	3.3 ± 3.1	3.0 ± 2.2	0.553
Freeze all oocytes or embryos	64.4% (65/101)	55.4% (56/101)	0.196
Number of transferred embryos	1.8 ± 0.4	1.8 ± 0.4	0.773

The data are presented as means ± standard deviations or percentages and counts. COS, controlled ovarian stimulation; G, gonadotrophin; E_2_, Estradiol; IVF, in vitro fertilization; ICSI, intracytoplasmic sperm injection; 2PN, 2 pronuclei.

**Table 3:** Linear regression model for the number of good-quality embryos.

Variable	Coefficient	95% confidence intervals		*p* value
		Lower limit	Upper limit	
SARS-CoV-2	0.161	-0.451	0.772	0.605
Duration of infertility	-0.153	-0.269	-0.038	0.009
AMH	0.156	-0.019	0.332	0.081
Number of oocytes retrieved	0.207	0.144	0.270	<0.001

### Cycle outcomes after fresh ET

In total, nine asymptomatic women with uninfected partners, four asymptomatic women with infected partners and two uninfected women with infected partners underwent fresh ET after thorough medical counselling. The clinical pregnancy rate did not differ (53.3% vs. 51.5%, *P* = 0.907) between the study and control groups. The two groups had similar rates of implantation, miscarriage, ectopic pregnancy and live birth (**Table 4**). In control group, a woman with a twin pregnancy delivered at 26 weeks due to an unavoidable miscarriage and a complication of SARS-CoV-2 infection. All of the other women had singleton pregnancies and delivered after 37 weeks without acquiring a new SARS-CoV-2 infection. Except for two term neonates from the control group who were admitted to the neonatal intensive care unit (NICU) for respiratory distress and hypoglycemia, the Apgar scores of all neonates in the infected and control groups at 1 and 5 minutes were 10. The pregnancy rate was not affected by SARS-CoV-2 infection according to the logistic regression model (*P* = 0.206).

**Table 4:** Pregnancy outcomes of fresh embryo transfer in the SARS-CoV-2-positive group versus the control group.

Variable	SARS-CoV-2 positive (n = 15)	SARS-CoV-2 negative (n = 33)	*P* value
Implantation rate	33.3% (9/27)	33.3% (20/60)	1.00
Clinical pregnancy rate	53.3% (8/15)	51.5% (17/33)	0.907
Miscarriage rate	12.5% (1/8)	25.0% (3/17)	0.743
Ectopic pregnancy	12.5% (2/8)	11.8% (2/17)	0.958
Live birth rate	33.3% (5/15)	39.4% (13/33)	0.688

### Cytokine profiling of asymptomatic or mildly symptomatic COVID-19 patients

**Figure 1:**
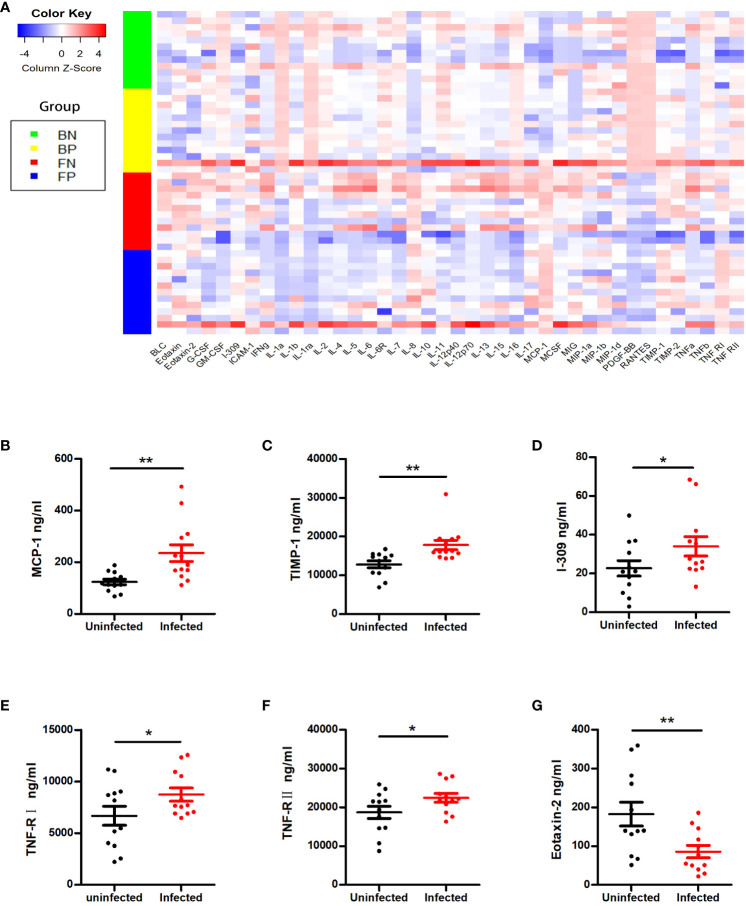
Cytokine alterations between the SARS-CoV-2-positive group and the SARS-CoV-2-negative group. **(A)** The Z scores of cytokines in the two groups. BN = the blood of the SARS-CoV-2-negative group; BP = the blood of the SARS-CoV-2-positive group; FN = the follicular fluid of the SARS-CoV-2-negative group; FP = the follicular fluid of the SARS-CoV-2-negative group. **(B–G)** Representative cytokines that showed significant differences between the two groups, including MCP-1 **(B)**, TIMP-1 **(C)**, I-309 **(D)**, TNF-RI **(E)**, TNF-RII **(F)** and eotaxin-2 **(G)**. *P < 0.05; **P < 0.01.

**Figure 2:**
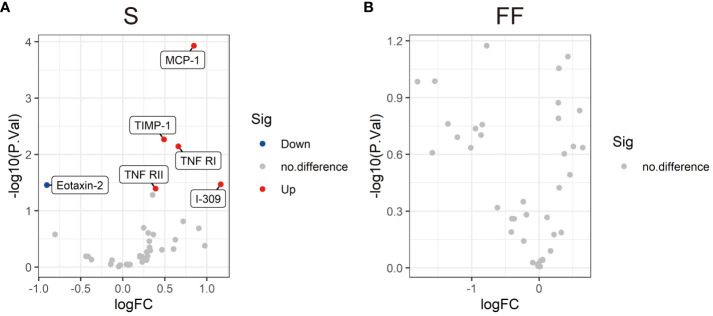
Volcano plots showing the differential levels of cytokines (DLCs) in serum **(A)** and follicular fluid **(B)** between the SARS-CoV-2-positive group and the SARS-CoV-2-negative group. Each dot represents a kind of cytokine, and the numbers of DLCs are indicated. Cytokine differential level analysis between the two different groups was performed by R version 4.3.2 software. The cut-offs for the DLCs were *P* < 0.05 and an absolute fold change >1.2.

To further check whether the changes in the cytokine profiles were associated with asymptomatic or mild COVID-19, we measured the serum and follicular fluid concentrations of 40 inflammatory factors in 13 female COVID-19 patients and 12 female non-COVID-19 patients (the study and control groups). In the infected group, the serum levels of monocyte chemoattractant protein-1 (MCP-1), tissue inhibitor of metalloproteinases 1 (TIMP-1), I-309, tumour necrosis factor receptor I (TNF-RI) and TNF-RII were higher than those in the uninfected group, while the serum concentrations of eotaxin-2 were lower (**Figures 1A–G**, **2A**). Further comparisons of the follicular fluid concentrations of 40 inflammatory factors were performed. No significant differences were observed between the two groups (**Figure 2B**).

## Discussion

This study is the first to examine the pregnancy rate and cytokine levels of patients infected with SARS-CoV-2 during controlled ovarian stimulation (COS). Our findings indicated that SARS-CoV-2 infection during COS did not negatively affect the pregnancy rate. SARS-CoV-2 infection did not impact embryo quality, number of retrieved oocytes, fertilization method, or rate of fertilization or live birth. Except for those of serum MCP-1, TIMP-1, I-309, TNF-RI, TNF-RII and eotaxin-2, the cytokine levels of asymptomatic and mildly symptomatic individuals infected with SARS-CoV-2 during COS were not significantly different. In addition, 40 inflammatory factors were compared in follicular fluid. No significant differences were found between the asymptomatic or mildly symptomatic COVID-19 patients and the uninfected controls. These findings indicated that the absence of symptoms or mild symptoms of SARS-CoV-2 infection did not have a negative impact on ART outcomes.

At the onset of the pandemic, the European Society of Human Reproduction Embryology (ESHRE) and the American Society for Reproductive Medicine (ASRM) advised halting fertility treatments. The purpose of these recommendations was to safeguard the well-being of couples undergoing ART and their newborns (22). Due to the lack of substantial evidences on the safety of SARS-CoV-2 infection during ART and concerns about the potential adverse effects of SARS‐CoV‐2 infection, women who were infected with SARS-CoV-2 were more likely intended to give up their cycles rather than continue during the early pandemic period (23).

During the epidemic, however, unplanned SARS-CoV-2-related closures occurred. Barragan et al. reported that oocytes from two women with asymptomatic SARS-CoV-2 infection showed no presence of SARS-CoV-2 RNA (11). The RNA of SARS-CoV-2 virus was not detectable in the follicular fluid, cumulus cells or endometrium of the infected women (24). Furthermore, a recent study revealed that eight individuals who were confirmed to have COVID-19 on the day of oocyte retrieval did not have SARS-CoV-2 RNA in their follicular fluid (25). It is possible that the zona pellucida could serve as a natural barrier for oocytes *in vivo* against SARS-CoV-2 infection (and other pathogens), despite oocytes and ovarian tissue expressing ACE2 and transmembrane serine protease 2 (TMPRSS2), to allow viral entry (26). According to these previous studies, one can be reassured regarding the possibility of contamination in ART laboratories. However, data regarding the impact of SARS-CoV-2 infection on the ART outcomes remains insufficient.

Youngster et al. conducted a retrospective cohort study on SARS-CoV-2-infected females who received ART within a year of infection (27). According to the study, the presence of SARS-CoV-2 infection did not have any impact on oocyte yield, fertilization or maturation, the number of high-quality embryos, or the rate of clinical pregnancy (27). Nevertheless, the duration between SARS-CoV-2 infection and oocyte retrieval ranged from 8 to 348 days. Similarly, Chen et al. reported that COVID-19 infection within a week prior to oocyte retrieval did not affect the development of oocytes and embryos (28). In contrast, Tian et al. discovered that patients with SARS-CoV-2 infection during COS had lower rates of top-quality embryos and blastocysts (29). Unfortunately, the pregnancy rate and severity of COVID-19 were not mentioned in that study. We observed no negative impact of SARS-CoV-2 infection on ART outcomes in asymptomatic or mildly symptomatic COVID-19 patients indicating that SARS-CoV-2 infection has no adverse effects on the development of oocytes/embryos or pregnancy. Previous studies on the effects of SARS-CoV-2 infection on semen quality have focused mostly on men who have recovered from the infection. Some studies have reported that a history of SARS-CoV-2 infection negatively affects semen parameters (30–32). However, semen parameters do not seem impaired after a mild infection (33). Our results indicated decreased semen volume, sperm concentration and total sperm in the infected male partner. However, the fertilization method and fertilization rate were not influenced. Due to the limitations of sample size, more studies with lager sample sizes are needed.

Systemic inflammation, which is commonly linked to acute COVID-19, can indirectly impact reproduction (34). Previous studies have shown that the severity of COVID-19 is linked to the levels of interleukin(IL)-2, IL-6, IL-8, and tumour necrosis factor-α (TNF-α), and alterations in the expression of these inflammatory cytokines may occur in the early stages of SARS-CoV-2 infection (12, 35, 36). Increased production and elevated levels of IL-6 are thought to be central to the development of the cytokine storms (37). At the same time, cytokines and hormones interact in a complex and systemic manner, influencing the development of follicles and pregnancy. Understanding the immune response of the host is essential for assessing the potential reproductive harm caused by SARS-CoV-2 infection. However, the alterations in cytokine levels in the serum and follicular fluid of patients infected with SARS-CoV-2 during ART treatment remain unknown. When we examined markers of overall inflammation in the blood of these COVID-19 patients, we noted that the serum and follicular fluid cytokine levels in both groups were similar, except for a few proinflammatory cytokines in the serum. The levels of the key proinflammatory cytokine MCP-1 were increased in the serum. MCP-1 can induce the luteolysis of the corpus lutea, regulate monocyte differentiation and play a role in cytokine production (17, 38). Since the cytokine storm did not occur at the early stage of infection, the subsequent immune changes induced by MCP-1 might be the key factor associated with the severity of COVID-19. Changes in TNF will alter the local cytokine balance, inhibit steroidogenesis, and result in miscarriage (39). However, except for the high serum TNF-RI and TNF-RII levels, the levels of TNF-α and TNF-β in both the serum and follicular fluid of the infected women did not differ. Since studies indicate the role of TNF receptors as possible blockers of TNF cytokine action (40), the higher receptor level might be related to the limited immune response. As shown in a prior study (41), even a lower level of chemokine eotaxin-2 in infected individuals might also be associated with a milder immune response. Compared to those in systemic immunity, cytokines in follicular fluid act directly on oocytes which is likely more important for modulating the reproductive processes. Aberrant cytokine level in follicular fluid can lead to abnormalities in folliculogenesis, oocyte quality and embryo developmental capacity (42). Specifically, the cytokine profiles of follicular fluid obtained from asymptomatic or mildly symptomatic COVID-19 patients were similar to those of the individuals in the control group.

The mild immune response may have contributed to the inclusion of patients with only mild clinical symptoms or asymptomatic patients. On the other hand, the COVID-19 vaccine could result in a large reduction in the incidence of symptomatic or severe COVID-19 disease (43). It may also play an important role in the reduced immune response.

The findings of our study indicated that mild or asymptomatic SARS-CoV-2 infection during COS did not have a detrimental impact on ART outcomes. This might be related to the absence of inflammasome activation observed in the follicular fluid of individuals with SARS-CoV-2 infection. In particular, subsequent changes in cytokine levels in infected couples may also be important for pregnancy outcomes and warrant further investigation.

The present study has several limitations. First, our research included only mild or asymptomatic individuals, and COVID-19 patients with moderate or severe symptoms were not included. Second, the analysis did not consider the distinct impacts of male or female infection ART outcomes of due to the small sample size. To ensure the reliability, future studies with larger cohorts and extended follow-up periods will be required for safety validation. Third, the cytokine profiles of pregnancy COVID-19 patients should be monitored. Finally, it was unknown whether COVID-19 infection during COS affects frozen ET outcomes.

## Conclusion

In conclusion, asymptomatic or mild COVID-19 during COS had no adverse effects on the outcomes of ART. Although mild inflammation was present in the serum, it was not detected in the follicular fluid of these patients. The subsequent immune response requires further investigation.

## Data availability statement

The raw data supporting the conclusions of this article will be made available by the authors, without undue reservation.

## Ethics statement

The studies involving humans were approved by The Ethics Committee of the Women’s Hospital, School of Medicine, Zhejiang University. The studies were conducted in accordance with the local legislation and institutional requirements. The participants provided their written informed consent to participate in this study.

## Author contributions

XH: Conceptualization, Data curation, Funding acquisition, Methodology, Project administration, Writing – review & editing, Resources, Software, Writing – original draft. GF: Conceptualization, Data curation, Funding acquisition, Methodology, Project administration, Resources, Software, Writing – original draft, Writing – review & editing. QC: Data curation, Project administration, Resources, Writing – original draft, Formal analysis. YS: Data curation, Formal analysis, Project administration, Resources, Writing – original draft. QQC: Data curation, Formal analysis, Project administration, Resources, Writing – original draft, Validation. SW: Data curation, Formal analysis, Project administration, Resources, Validation, Writing – original draft. SL: Data curation, Formal analysis, Project administration, Resources, Writing – original draft. LB: Data curation, Formal analysis, Project administration, Resources, Writing – original draft, Validation. YZ: Data curation, Project administration, Conceptualization, Funding acquisition, Methodology, Supervision, Writing – review & editing.
