# Supplementary material for: The impact and inflammatory characteristics of SARS-CoV-2 infection during ovarian stimulation on the outcomes of assisted reproductive treatment
Source: Front Endocrinol (Lausanne). 2024 Apr 25;15:1353068. doi: 10.3389/fendo.2024.1353068 (PMC11079226; doi:10.3389/fendo.2024.1353068)
Supplement: Supplementary file 1 [file Table_1.docx]

Table S1. Sperm parameters of SARS-CoV-2-infected male partners pre- and post-SARS-CoV-2- infection.

| **Semen parameters** | **Pre infection** | **Post infection** | ***P* value** |
| --- | --- | --- | --- |
| Semen volume (ml) | 3.3±1.1 | 2.1±0.5 | <0.001 |
| Sperm concentration (10^6^/ml) | 69.2±43.8 | 59.86±29.4 | 0.002 |
| Total sperm count (10^6^ ) | 227.0±179.7 | 130.0±74.3 | <0.001 |
| Progressive motility (%) | 42.60±16.8 | 33.92±16.8 | 0.697 |
| Total motility (%) | 53.5±19.4 | 45.2±18.3 | 0.551 |
| Immotile sperm (%) | 46.5±19.4 | 54.78±18.3 | 0.551 |
